# Caregiver policies in the United States: a systematic review

**DOI:** 10.1057/s41271-024-00529-7

**Published:** 2024-11-17

**Authors:** Makenna R. Green, M. Courtney Hughes, Sadia Afrin, Erin Vernon

**Affiliations:** 1 School of Interdisciplinary Health Professions, Northern Illinois University, DeKalb, IL USA; 2 Department of Public Health, Northern Illinois University, DeKalb, IL USA; 3 Department of Economics, Seattle University, Seattle, WA USA

**Keywords:** Caregiver support, Informal caregiving, Public policy, Serious illness

## Abstract

**Abstract:**

In the United States, there are nearly 53 million informal or unpaid caregivers, many of whom experience mental and physical stress related to their caregiving duties and increased financial responsibility. We identified federal and state informal caregiver support policies authorized by specific legislation along with their key provisions and conducted a systematic review of the academic literature related to quantitative evaluations of these policies. Twenty policies, eight academic studies, and four gray literature reports were included in the study, with half of the policies introduced since 2000. Our study criteria yielded few academic valuations tied to caregiver policies and few policies including research provisions. Of the provision areas identified in policies, respite services, caregiver training, and workplace protections appeared the most. Future policies and the studies examining them should incorporate cost outcomes and equity as focus areas and disaggregate data by vulnerable groups to ensure value and equity in caregiver support legislation.

**Key messages:**

- Increased legislation to support informal caregivers may be warranted. The limited academic research examining existing caregiver policies identifies mixed outcomes for caregivers.
- Prioritizing vulnerable populations in such policy research examining outcomes could help improve caregiver support efforts.
- The included studies investigated the outcomes of three policies and identified more negative than positive outcomes for caregivers.

**Supplementary Information:**

The online version contains supplementary material available at 10.1057/s41271-024-00529-7.

## Introduction

Informal caregivers (“caregivers”) are friends, family, or others who assist individuals with daily activities or medical care due to illness, disability, or age-related issues and are unpaid for their services [1]. An estimated 53 million caregivers in the United States enable adults needing assistance to maintain their independence and avoid costly care, such as nursing homes [2–4]. As adults in the United States (US) increasingly suffer from chronic diseases, live longer with such diseases, and exist in a health system plagued by provider shortages, the burden placed on caregivers is projected to continue its growth. Caregivers provide a range of services from supporting basic activities of daily living such as dressing and grooming their loved one to performing wound care, managing feeding tube sites, and other medical tasks. Consequently, the economic value of these unpaid caregiving services will transcend its current annual cost of $600 billion [5, 6].

Caregiving work includes difficult tasks such as helping to manage pain and symptoms, keeping track of medicine schedules, transporting sick individuals to appointments, and providing emotional support. Not surprisingly, caregivers suffer from higher rates of depression and anxiety and miss more workdays than the non-caregiver population [2]. While a myriad of different policies which aim to help patients can indirectly benefit their caregivers, many government policies have specifically targeted support for caregivers in their challenging roles.

We aim to provide the first systematic review of the literature to date focusing on federal and state caregiver support policies in the United States, highlighting the potential economic and equity impact. First, we examine the existing policies and then investigate the related academic literature. We specifically report on outcome measures, policy effectiveness, cost impact, and consideration of vulnerable populations (groups at higher risk for poor health care outcomes or lacking access to health services). While policies which focus on care provision, housing and transportation can positively impact family caregivers, understanding the impact of unpaid caregiver targeted policies can help further inform future government initiatives to improve the situation for caregivers in the United States.

## Methods

This systematic review was registered at PROSPERO (CRD42022358599). We followed the Preferred Reporting Items for Systematic Reviews and Meta-analyses (PRISMA) reporting guidelines [7].

### Policy search strategy, criteria, and data extraction

We conducted a review study to investigate the topic of federal and state policies which specifically focus on caregiver support through 1 October 2022. To gather pertinent sources, we conducted a comprehensive search of Congress.gov and Google using various keyword strings, including”caregiver” or “caregiving” with each of the following terms: “policies,” legislation,” and “bill.” The study has been registered with PROSPERO (CRD42022358599).

We completed extraction of outcome and evaluation data for each included policy located via a systematic search on Google. We utilized the search string “policy name” and “outcomes.” If no novel results were yielded via the search strategy the search term “evaluation” was used. The authors completed title screening to determine relevance to the search terms. We then reviewed relevant results for keywords “outcomes” and “evaluation” to determine the presence and to what extent outcome measurement was employed. Data regarding policy outcomes is included in Supplementary Materials Table S1. For conciseness and brevity, we chose not to separately include state-level programs that were funded by larger national policies, such as the National Family Caregiver Support program (Older Americans Act Title IIIE).

First, we removed any duplicated policies. Then, at least two independent study authors reviewed the remaining policies based on predetermined inclusion criteria. We included all introduced federal and state bills that were formulated through the legislative process and related to supporting informal or family caregivers. We excluded policies that were only focused on paid caregivers; including paid caregivers includes health care professionals and is beyond the scope of this review. Further, policies that were not formed with the informal caregiver as the direct recipient of benefit were excluded from this study. After screening the policies, at least two study authors independently obtained and evaluated detailed policy information for inclusion. Any discrepancies or disagreements during the inclusion process were resolved through discussion among the study authors. This comprehensive screening process ensured that we captured a wide range of relevant policies and perspectives on the topic.

Each policy that met the inclusion criteria underwent independent review by at least two study authors who extracted key information, including the policy's name, type (national or state), status (introduced, established, or amended), year of introduction and amendment, eligibility criteria, benefits, and implications. Furthermore, the authors extracted information related to the policy's purpose and focus areas.

### Publication search strategy, criteria, data extraction, and quality assessment

We conducted a systematic review of literature related to caregiver supports and policies through 1 October 2022. We searched PubMed (including MEDLINE), CINAHL, PsychINFO, and ProQuest Federated databases and conducted a comprehensive web search to gather relevant studies. We used the same keyword search string as for the existing policies to identify relevant articles, which were then screened based on the presence of these keywords in the title or abstract. To determine our keywords, we followed the guidance of Bramer and colleagues’ [8] guidance on efficiently completing literature review searches.

Duplicate studies were first removed, and then at least two study authors independently assessed the remaining abstracts based on predetermined inclusion criteria. Studies had to examine the impact of enacted federal or state policies in the United States related to supporting informal or family caregivers. We excluded policies that were only focused on paid caregivers; including paid caregivers includes health care professionals and is beyond the scope of this review. We considered all quantitative study designs and articles in different languages. We excluded nonprofit or company-run caregiver support policies that were not governmental policies formulated through the legislative process. Full-text articles were independently evaluated for inclusion by two study authors, with discrepancies resolved through discussion.

For each study that met the inclusion criteria, at least two study authors extracted information independently, including the study aim, policy name, type (national or state), eligibility, benefits, study design, whether vulnerable populations were included, costs, and the study's findings. The authors discussed and resolved any discrepancies in the extracted data.

The quality of the evidence for each study was assessed using the criteria from the Oxford Centre for Evidence-Based Medicine [9]. Two study authors independently graded the strength of each study, with any discrepancies resolved through discussion. The quality ratings were assigned as follows: rating 1 for properly powered randomized clinical trials and systematic reviews with meta-analysis, rating 2 for well-designed controlled trials without randomization and prospective comparative cohort trials, rating 3 for case–control studies and retrospective cohort studies, rating 4 for case series with or without intervention and cross-sectional studies, and rating 5 for case reports or opinions of respected authorities.

As suggested in the gray literature research guidelines [10], the study authors searched the National Technical Reports Library [11], Google with limitations for government documents, and the AARP research database [12]. For these databases, the researchers searched for the term “caregivers” in title documents. Reports and articles matching the researchers’ selection criteria underwent the same data extraction processes as the academic studies.

## Results

### Existing policies

Electronic web searches resulted in 162 retrieved policies related to supporting informal or family caregivers. After initial screening by two independent reviewers, 120 policies were removed. Exclusion reasons included lack of relevance to informal caregivers, amendments as opposed to the original legislation, and programs only that were not formulated through the legislative process. We removed an additional 15 policies because they were not enacted and one policy because it was not funded (Supplementary Materials Figure S1). Of the 20 analyzed policies, 45% (*n* = 9) were federal policies, 15% (n = 3) were state-level policies enacted in more than 10 states, and 40% (*n* = 8) were state-level policies in single states (Supplemental Materials Table S1**).** Populations that policies targeted varied, with one-quarter of the policies specifically focusing on the care of older adults. In total, 25% (*n* = 5) of the policies have been updated in the last 10 years, adding additional provisions or funding. Our examination of outcome measurements showed that some federal policies that disseminate funding to states have outcome measurement requirements built into the body of their legislation. Outcome evaluation is specified in 30% (n = 6) of the examined policies’ legislation. The administrator (agency disseminating program provisions or funding) evaluated another 25% (*n* = 5) of policies despite it not being a requirement, most often at the state level. Outcomes were not evaluated in 40% (*n* = 8) of the policies. A single policy (5%) implied outcome evaluation in its legislation but included no formally specified measures. We also found that these requirements are often broad and do not specify how data are collected and distributed to the public. (Note, the above description of evaluation being part of legislation is not related to our author group evaluating policies for this paper.) Further details about all policies, including the 16 that were not passed or funded, are in Supplementary Materials Table S1.

The 20 included policies crossed several categories of provision and function. Examining each policy’s primary purpose yielded eight focus areas (Table 1)**.** Respite services were well-represented in the policies, with seven focusing on establishing or supporting these services. Most policies addressing respite services also included other provisions within the legislation, such as caregiver training or care coordination. The majority also include other provisions that aim to improve the lives of caregivers overall. For example, one such policy, the Pennsylvania Caregiver Support Act, provides services across half of the policy focus areas.

**Table 1:** Policy focus areas: definitions and counts

Policy focus area	Definition	Number of national policies (%)	Number of state policies (%)	Total number of policies (*n* = 20) (%)
Respite services	Provides short-term relief to caregivers	3 (43)	4 (57)	7 (35)
Caregiver training	Supports the education and training of caregivers on topics related to their role as a caregiver	3 (50)	3 (50)	6 (30)
Workplace protections	Provides employees with financial, disciplinary, or work schedule safeguards for caregiving	2 (33)	4 (67)	6 (30)
Care coordination	Facilitates development of care pathways and services that benefit caregivers	2 (40)	3 (60)	5 (25)
Payment for caregiver needs and services	Provides direct or indirect monetary compensation for caregivers for needed or provided services	1 (25)	3 (75)	4 (20)
Caregiver advocacy	Promotes and facilitates the communication of caregivers’ experiences, views, and needs	2 (67)	1 (33)	3 (15)
Counseling services	Provides mental health services to caregivers	2 (67)	1 (33)	3 (15)
Research	Provides funds directly to support quantitative analysis of caregiver issues	1 (50)	1 (50)	2 (10)

Like respite services, caregiver training is common among sweeping legislation to assist caregivers holistically. Six policies address caregiver training, with none targeting training in isolation. Over half of the policies addressing caregiver training are enacted at the state level [13-15-12]. Two of the three national policies addressing caregiver training are strictly for U.S. veterans and their family members who are eligible to receive benefits through the U.S. Department of Veterans Affairs [16, 17]. One national level policy provides caregiver training services to the general public (National Family Caregiver Support Program). Additionally, five policies aim to improve care coordination.

Six policies include workplace protections, one of which was enacted in 10 states and guarantees partial or full wage compensation during caregiving without accruing time off work [18]. Two other workplace protection policies provide job protection for caregiving during periods of leave for up to 12 weeks with no guaranteed compensation [19, 20]. Another type of workplace protection benefit found in two policies targets sick leave time and employees’ rights to accrue paid sick time that can be used for caregiving [21, 22]

The remaining areas include caregiver counseling services, payment for caregiver needs, caregiver advocacy, and research. Counseling services include provisions to reduce the mental health ramifications of informal caregiving. One of the three policies with counseling services is a national level policy available to the general public, the National Family Caregiver Support Program. Additionally, payment for caregiver needs and services also represents four of the 20 policies examined. Of these four policies, one policy provides a stipend to caregivers for their services [16]. This policy provides payments on a tiered system based on the need level of the care recipient/veteran. The remaining three policies, both operating at the state level, stipulate that payments are reimbursement for services related to caregiver need or equipment. The remaining two focus areas, caregiver advocacy (improving community awareness and facilitating action regarding caregiver needs) and research, have three and two policies, respectively, that fall within their provisions. Both caregiver advocacy and research related to caregiver support are secondary benefits in their bills. Two of the analyzed policy focus areas were targeted more often by national legislation, four by state-level legislation, and two had an even distribution (see Table 1).

### Evaluative records

After screening abstracts, we assessed 159 records for eligibility. After applying inclusion and exclusion criteria, 12 records were selected for inclusion (Supplementary Materials Figure S2). Table 2 summarizes the eight policies included in the academic studies and provides the outcomes these studies examined and the reported results. Similarly, Table 3 displays the policies examined within the 4 included gray literature records. Table 4 presents the characteristics of all the included records. Regarding the quality of the evidence, all the records were rated in the bottom three (of five) categories, with most in the second to lowest category indicating the research design for these studies was not consistently of high quality. The records are further summarized in Supplementary Materials Table S3.

**Table 2:** Outcomes by policies examined in the academic literature

Outcomes	Success for caregivers (Yes/No)^1^
National Family Caregiver Support Program [23–28]	
General	
Eligibility requirements do not allow the availability of services to the majority of caregivers in need (Litzelman, 2022)	No
No consistent, standardized outcome evaluations exist to examine the program’s impact on caregivers and care recipients (Shugrue, 2019)	No
Negative association of policy and unused services (Potter, 2018)	Yes
No association between funding and all services used (Potter, 2018)	No
A state’s history of supporting caregivers as both service recipients and providers predicts the likelihood of more counseling, support groups, and training delivery (Giunta, 2010)	Yes^a^
Caregiver financial assistance in the US falls short compared to other countries (Whittier, 2005)	No
Multiple forms of caregiver service delivery in existence (Whittier, 2005)	Yes
Equity-related	
Caregivers of Black and Hispanic older adults were more likely to report any unused services (Potter, 2018)	No
Hispanic caregivers reported that 43.4% of care recipients received fewer hours of respite care than their estimated need, compared with 32.2% of African Americans and 28.4% of Whites (Herrera, 2013)	No
Hispanic caregivers were less likely to report that supportive services were paid for by the care recipient or other family members (Herrera, 2013)	Yes
Volume of caregiver and other support services used, receiving amount of hours of respite care based on need, and living independently due to benefits were not the same across races (Herrera, 2013)	No
When comparing the risk profile of caregivers receiving policy-related services to the general population of seniors, services are reaching the most vulnerable populations according to most risk factors for institutionalization, such as disability, Medicaid coverage, and living alone (Herrera, 2013)	Yes
Caregiver service gaps not closed. (Service gaps most frequently identified included culturally and linguistically appropriate caregiver services, transportation, respite care, financial assistance, and services in rural areas.) (Whittier, 2005)	No
There was overrepresentation of high-risk subsets of caregivers, including low-income caregivers, among those deemed ineligible for benefits (Whittier, 2005)	No
Caregivers and Veterans Omnibus Health Services Act of 2010 (Caregiver Support Line) [30]	
General	
High caller satisfaction (Wright, 2015)	Yes
High utilization rates (Wright, 2015)	Yes
Equity-related	
Use in rural areas is proportional to the number of veterans living in rural areas (Wright, 2015)	Yes
California’s Family Rights Act (Paid Leave Law) [31]	
General	
No effects on caregiver mental or physical health (Gimm, 2016)	No

^a^Finding helps understanding of successful caregiver policy implementation

^1^Yes/No success for caregivers refers to the authors appraisal of the included studies reported outcomes for caregivers and if they positively benefited caregivers

**Table 3:** Outcomes by policies examined in the gray literature

Outcomes	Success for caregivers (Yes/No)
Family Medical Leave Policies [32]	
General	
1 in 3 workers is unaware of FMLA benefits (AARP, 2013)	No
37% of employers report positive effect and 54% report no noticeable effect on absenteeism, turnover, and morale (AARP, 2013)	Yes
54% of workers do not receive any pay while on leave (AARP, 2013)	No
88% of workers age 50 + found FMLA protections important to them (AARP, 2013)	Yes
12% of workers have access to paid family leave (AARP, 2013)	No
Paid leave benefits to employer and employee with consistent workforce participation and improved quality of life (AARP, 2013)	Yes
12% of workers have access to paid family leave (AARP, 2013)	No
Paid leave benefits to employer and employee with consistent workforce participation and improved quality of life (AARP, 2013)	Yes
99% of employers reported a positive or no noticeable effect on turnover, productivity or performance (AARP, 2013)	Yes
Employees reported increased ability to care for family member and increased likelihood of returning to work following leave (AARP, 2013)	Yes
Low public knowledge of paid leave benefit (AARP, 2013)	No
Equity-related	
80% of low wage workers do not have access to paid sick days (AARP, 2013)	No
Older Americans Act [33]	
General	
National Family Caregiver Support Program is effective in reducing caregiver burden (AARP, 2019)	Yes
Caregivers who receive 4 or more hours of respite care a week reported decline in burden (AARP, 2019)	Yes
Caregivers who receive at least one education, counseling, or support group session reported improved confidence (AARP, 2019)	Yes
Caregivers provided $470 billion in unpaid care in 2013, but only $181 million of the Older Americans Act is allocated to caregiver support services (AARP, 2019)	No
Equity-related	
Older Americans Act funding is approximately $2 billion (comparatively, Medicaid waiver funding is $167 billion), findings suggest middle class caregivers ineligible for many supports (AARP, 2019)	No
Recognize, Assist, Include, Support, & Engage (RAISE) Family Caregivers Act [34]	
General	
Access and presences of Direct Support Professionals (e.g., CNA’s, home health aids) have not been sufficient in supporting the needs of family caregivers (ACL, 2021)	No
Lack of representation of caregiver perspectives in policy and healthcare systems (ACL, 2021)	No
Development and implementation of evidence-based caregiver assessment tools (e.g., BRI Care Consultation and Tailored Caregiver Assessment and Referral) (ACL, 2021)	Yes
Considerable variability in how “caregiver” and “support” is defined across government agencies that may support caregivers (ACL, 2021)	No
Lack of caregivers in research efforts or data collection (ACL, 2021)	No
National Family Caregiver Support Program/Medicaid Home and Community Based Services waivers/ State funded actions [29]	
General	
36% of states began providing support to caregivers of older people for the first time (Family Caregiver Alliance, 2004)	No
Area Agencies on Aging are the most common agency to organize caregiver support programming (Family Caregiver Alliance, 2004)	Yes
High variability in how states track program expenditures and service delivery (Family Caregiver Alliance, 2004)	No
Multiple funding sources reported across states with main sources including: State general funds, NFSCP, Medicaid HCBS waivers, and client contributions (Family Caregiver Alliance, 2004)	No
Less than half of the programs included uniformly assess caregiver needs (Family Caregiver Alliance, 2004)	No
Respite is most common service offered and is available across all states though with great variability (Family Caregiver Alliance, 2004)	Yes
Access to program information or services vary by program type (Family Caregiver Alliance, 2004)	No
Caregiving is assessed in only 5 states’ uniform assessment tool of HCBS programs (Family Caregiver Alliance, 2004)	No
States identify inadequate funding as main barrier to implanting caregiver programs (Family Caregiver Alliance, 2004)	No

**Table 4:** Characteristics of the evaluative records^a^

	Number of evaluative studies n (% of 12 studies)
Record quality	
1 (Randomized controlled trial)	0 (0.0)
2 (Non-randomized controlled trial; prospective comparative cohort trial)	0 (0.0)
3 (Case–control studies; retrospective cohort study)	2 (16.7)
4 (Case series with or without intervention; Cross-sectional study)	6 (50.0)
5 (Opinion of respected authorities; case reports)	4 (33.4)
Treatment of costs	
Examined costs	4 (33.4)
Mentioned costs	6 (50.0)
Did not mention costs	2 (16.7)
Vulnerable populations examined	
Socioeconomic status level	5 (41.7)
Rural areas	2 (16.7)
Caregivers of Black and Hispanic older adults	3 (25.0)
None	4 (33.4)

^a^ Percentages might not add to 100% due to rounding or, in the case of vulnerable populations examined, studies included multiple categories

The six academic studies on the NFCSP were all cross-sectional [23–28] study designs and found more negative than positive outcomes for caregivers [23–28]. Half of the 14 caregiver-related outcomes examined for this policy were related to equity, with the majority indicating a lack of equity for vulnerable racial and low-income groups [25, 26, 28]. Specific benefits mentioned as not being used as much by vulnerable populations included respite care, [25, 26, 28] a lack of culturally and linguistically appropriate caregiver services, transportation, and financial assistance [28]. Whittier et al., [28] also found that service gaps for caregivers existed in rural areas. One study on the NFCSP reported costs, calculating that other countries perform better than the United States in financially supporting caregivers [28]. Shugrue and colleagues (2019) reported the need for consistent, standardized outcome evaluations of NFCSP. A gray literature report related to this policy also suggested a lack of consistent assessment of policy as well as funding across states [29].

The two academic articles that didn’t examine NFCSP were both cohort studies. The study examining the Caregivers and Veterans Omnibus Health Services Act of 2010 reported high caller satisfaction and utilization of a caregiver support line [30]. The study examining the California Family Rights Act’s Paid Leave Law used nationally representative panel data from eight waves of the Health and Retirement Survey (HRS) and reported that caregiver mental and physical health did not improve as a result of the policy [31]. Additionally, the academic articles lacked a cost outcomes focus, with three-quarters of the articles not analyzing costs.

The three remaining gray literature records assessed Family Medical Leave Act (FMLA) policies, the Older Americans Act, in general, and the RAISE Act. While recipients of FMLA benefits primarily reported positive outcomes, there were general knowledge and access gaps related to the policy [32]. A study of the Older Americans Act reported similar findings in that those receiving benefits reported improved outcomes; however, the report noted access to the benefits could be improved [33]. A report on the RAISE Act also highlighted access concerns and the lack of caregiver involvement in policy provision assessments [34].

## Discussion

This systematic review of existing caregiver policies in the United States and the academic literature about them revealed a growing number of such policies and a lack of scientific examination reporting their outcomes. Our analysis of caregiver policy provisions indicates that respite services, caregiver training, and workplace protections are the benefits most offered in the policies. A noticeable omission in existing caregiver policies was a focus on vulnerable populations beyond that of unpaid caregivers (who can be considered a vulnerable group in and of themselves). In our review of the academic literature, four of the six included studies that investigated equity-related outcomes. This represented just two of the 20 total included policies (NFSCP and the Caregivers and Veterans Omnibus Health Services Act of 2010) with most of those measures indicating that vulnerable populations were worse off than their counterparts in receiving or experiencing benefits from those policies. Given that today’s standards are more focused on measuring outcomes of groups in particular need of services, it is not surprising the studies from the past may not have centered on this aspect.

While our study shows that existing policies have attempted to help support caregivers, the growing prevalence of mental and physical health issues among caregivers [35] are a strong indication that more must be done. For example, seven existing policies help provide respite care, yet only 14% of a nationally representative sample of caregivers in the US report utilizing respite care. Similarly, three major federal policies [16, 17, 36] and three state policies [13–15] include caregiver training provisions. Yet, over half of a representative national sample of caregivers in the United States report lacking information in at least one area related to caregiving [35]. Given this repeated pattern of unmet needs in the presence of existing policies attempting to address them, more focus should be placed on benefits dissemination and improving policy effectiveness.

Only a few equity-related outcomes are presented in the academic literature appraising policies. This is not surprising given that the push to disaggregate research outcomes has been more recent, particularly coming to light during the COVID-19 pandemic when vulnerable populations experienced significantly worse health outcomes [37]. While not always feasible given data and resource limitations, examining policies’ impact on vulnerable groups can provide value given the documented support needs in these populations [38].

The need to disaggregate outcomes data from policies impacting caregivers will help address inequities in caregiver support [37]. Researchers should collect and attribute data from each minority racial group, trying not to use “catchall” categories, and based on other characteristics such as metropolitan status, income, education, and gender-related groups to which caregivers and the individual needing care belong. Not only should this data appear in evaluative studies about the policy, but it would be helpful for those evaluating policies or caregiver advocate groups to create data dashboards so that policymakers and other decision-makers can more easily explore outcomes of interest in a disaggregated format. It is important to note that collecting data from caregivers may be more challenging than collecting data from professional caregivers, who may have administrative staff who can help support data collection.

The financial toll of caregiving has been well documented, with half of a nationally representative group of caregivers reporting at least one type of financial impact due to unpaid caregiving. These financial impact types are defined by the AARP and NAC report (2020) as stopping saving, taking on debt, using personal savings, leaving bills unpaid, and borrowing money. Further, nearly 20% of caregivers report high financial strain due to caregiving [35]. The financial toll of caregiving also disproportionately impacts people of color, with Black caregivers reporting nearly double the financial impact than their white counterparts among those earning more than $50,000 a year in income. Moreover, caregivers often report that compensation for caregiving hours, paid leave, and tax credits would be beneficial [35]. Some enacted policies provide such benefits. It is also important to note that another avenue to supporting caregivers is policy that channels funds to improve home health services or long-term care. This alternative approach may mean fewer dollars go directly to caregivers. Future research should examine the optimal policies for adults who need care.

The presence of policies, both only introduced and enacted, has grown over the last decade. Numerous pieces of legislation supporting caregivers have been presented to Congress or state legislatures, with over half of caregiver support legislation introduced in the last 23 years being in the last three years alone. This trend aligns with the growing population of older adults and the increasing prevalence of caregivers caring for them. From 2015 to 2020, caregivers rose from 43.5 million to 53 million [35]. As recently as March of 2023, the Home and Community Based Services Access Act was proposed to amend Title XIX of the Social Security Act, which would provide support for caregiving costs and allow recipients to hire family members as their caregivers [39]. Although the increased caregiver legislation activity induces optimism, legislative efforts must continue to garner non-partisan support and keep up with the pace of caregiving needs burdening our nation.

A challenge with supporting caregivers through policies is that the policies must be fully funded. Our review covers policies supporting caregivers that have been passed. A future review should consider the percentage of evidence-based programs that do versus do not get implemented into state or federal policy. Ideally, such a review would also include the percentage of ancillary policies (such as meals and transportation) that are fully implemented. A body of caregiver support literature describes programs that can effectively aid caregivers [35], yet many such programs do not get implemented as policies. Furthermore, while passing caregiver policies can establish the importance of supporting caregivers, only when these laws are fully funded can they meaningfully impact caregivers’ lives.

Our finding that there is a lack of evaluation of caregiver outcomes resulting from caregiver policies is consistent with gaps found across other policy areas. The U.S. Government Accountability Office (GAO) conducts a review of all federal agencies’ evaluation procedures. The GAO found that 40% of agencies had conducted a review in the last five years, 39% did not know if a review had been conducted, and 18% reported no evaluation having been conducted [40]. The GAO suggests that this lack of evaluation is a missed opportunity to increase the effectiveness of government resources [40].

It is important to note that this review focused on informal caregivers, which, by nature, excludes informal caregivers who have become paid caregivers via Medicaid and other state-level programs. While such programs are beyond the scope of our focused review, emerging research into such programs suggests that they can be cost-neutral ways to improve patient outcomes, especially for underserved populations [41]. Future research should examine policies that include informal caregivers who are paid. Evidence exists regarding the relative paucity of caregiver support provisions and the potential for success, further supporting the notion that paying family caregivers through Medicaid or other consumer-directed programs can be an effective and essential piece of successfully supporting the needs of the increasing caregiver population in the United States [42]. We recommend a future systematic review that evaluates programs such as the Medicaid waiver programs by state, along with the academic studies and gray literature evaluating their outcomes for caregivers.

We summarized whether articles and reports found selected caregiver policies authorized by federal and state law to successfully impact the study team’s chosen variable(s) of study. This binary summary of article results (yes or no for success for caregivers) may have simplified some of the more nuanced findings from the research. In addition, due to the heterogeneity of the included articles and lack of consistent research design rigor, a meta-analysis was not undertaken for this systematic review. As for the summary of the policies themselves, the authors categorized their provisions into specific categories, which also had the potential to mask some of the more nuanced components of these multifaceted policies. A final limitation is that our review had low sensitivity with respect to detecting the presence or absence of policies in a given domain. For example, our study focused on caregiver policies and did not include ancillary policy areas such as care management, personal care, meals, transportation, and housing. Our study also did not include programs that were developed as part of enabling regulations or regulatory guidance. Adopting a policy that supports caregivers (or accessing such a benefit) relies on an ecosystem of supportive programs that generations of funding cuts and privatization have decimated. We recommend future research investigating these programs in more detail.

## Conclusions

This analysis of federal and state caregiver support policies in the United States and a systematic review of quantitative academic research examining their effectiveness highlight areas of potential improvement. The topic of caregiver policies lacks rigorous academic research, with researchers having quantitatively examined only a few of the policies. Our findings highlight the need for further research related to caregiver specific support policies.

## Supplementary Information

Below is the link to the electronic supplementary material.

Supplementary file1 (DOCX 98 KB)

## Data Availability

All data generated or analyzed during this study are included in this published article and its supplementary information files.
