# Supplementary material for: Caregiver policies in the United States: a systematic review
Source: J Public Health Policy. 2024 Nov 17;46(1):22–37. doi: 10.1057/s41271-024-00529-7 (PMC11893460; doi:10.1057/s41271-024-00529-7)
Supplement: Supplementary file 1 — Supplementary file1 (DOCX 98 KB) [file 41271_2024_529_MOESM1_ESM.docx]

**Caregiver Policies in the United States: A Systematic Review**

Makenna R. Green, MA^1^, M. Courtney Hughes, PhD, MS^2^,

Sadia Afrin^3^, Erin Vernon, PhD, MA^4^

^1^ School of Interdisciplinary Health Professions, Northern Illinois University, DeKalb, Illinois, USA

^2^ Department of Public Health, Northern Illinois University, DeKalb, Illinois, USA

^3^ Department of Public Health, Northern Illinois University, DeKalb, Illinois, USA

^4^ Department of Economics, Seattle University, Seattle, Washington, USA

**Supplementary Material**

**Figure S1**: PRISMA Flow Diagram for Policies

**Identification of policies via databases and registers**

Records identified from

Google (n = 136)

Government sites (n = 26)

Records removed before screening

Duplicate policies removed (n = 82)

**Identification**

Records screened

(n = 80)

Records excluded

(n = 38)

**Screening**

Reports not retrieved

(n = 0)

Reports sought for retrieval

(n = 42)

Reports assessed for eligibility

(n = 42)

Reports excluded:

Not about caregivers: (n = 6)

Policies not passed: (n = 15)

Policies not funded (n = 1)

Policies included in review

(n = 20)

**Included**

*From:*  Page MJ, McKenzie JE, Bossuyt PM, Boutron I, Hoffmann TC, Mulrow CD, et al. The PRISMA 2020 statement: an updated guideline for reporting systematic reviews. BMJ 2021;372:n71. doi: 10.1136/bmj.n71

**Figure S2**: PRISMA Flow Diagram for Evaluative Studies

**Identification of studies via databases and registers**

Academic studies from

PubMed (n = 700)

CINAHL (n= 452)

PsycINFO (n= 737)

ProQuest (n= 641)

Grey literature from

NTRL (n=167)

Custom Google (n=20)

AARP (n=268)

Duplicate records removed before screening (n = 1046)

**Identification**

Records excluded

(n = 1780)

Record abstracts and summaries screened (n = 1939)

**Screening**

Records excluded

Not about caregivers: (n = 10)

Not about caregiver policy: (n = 76)

Review: (n= 31)

Non-USA articles: (n= 5)

Not evaluative: (n= 25)

Records assessed for eligibility

Academic: (n = 132)

Grey Literature (n=27)

Records included in review

Academic: (n=8)

Grey Literature (n=4)

*From:*  Page MJ, McKenzie JE, Bossuyt PM, Boutron I, Hoffmann TC, Mulrow CD, et al. The PRISMA 2020 statement: an updated guideline for reporting systematic reviews. BMJ 2021;372:n71. doi: 10.1136/bmj.n71

**Included**

**Table S1.** **Enacted Policy Overview**

| **Policy Name** | **Year Introduced**  **/Passed or Updated** | **Location** | **Outcomes** | **Target Population** | **Policy Category** |
| --- | --- | --- | --- | --- | --- |
| Alabama Lifespan Respite Resource Network [1,2] | 2000/2016 | AL | No formal outcomes specified. Administrator measures current capacity and improvement of Alabama Respite Network as perceived by family caregivers, agencies, and providers; 2) identifies needed enhancements to respite worker training and service delivery; and (3) identifies facilitators and barriers to a coordinated system of lifespan respite care. | Caregivers of individuals with special needs | Respite Services; Care coordination |
| Alzheimer's State Plan [3] | 2016/2016 | WA | Legislation mentions measuring outcomes based on plan’s goals and informal needs surveys. | Alzheimer's care recipients, caregivers, and families | Caregiver Training; Respite Services; Care Coordination; Research |
| California Family Rights Act [4] | 1993/2022 | CA | No formal outcomes specified other than employment data collected. | Employees within CA | Workplace protections |
| Caregiver Advise, Record, Enable Act [5] | 2016/2019 | 42 States | No formal outcomes specified. | Hospitalized individuals and their caregivers | Care Coordination; Caregiver Training |
| Caregiver Leave Act [6] | 2016/2019 | NM | No formal outcomes specified. | NM employees | Workplace Protections |
| Caregivers and Veterans Omnibus Health Services Act of 2010 [7] | 2009/2010 | National | Formal outcomes specified include collecting demographic data, cost data, caregiver benefit, and services provided. | Caregivers of post-9/11 veterans and veterans with serious injuries or illnesses | Respite Services; Counseling Services; Caregiver Training; Payment for Caregiver Needs |
| Dependent Care Assistance Program [8] | 1986/2021 | National | No formal outcomes specified. | Employees with dependent care expenses while at work, like child or elder care | Workplace protections |
| Family and Medical Leave Act [9] | 1993/1993 | National | No formal outcomes specified. Administrators measure demographics, percentage of leave taken, knowledge of leave, employer dissemination of leave information. | Employees taking leave for caring for family members | Workplace protections |
| Kupuna Caregivers Program [10] | 2017/2018 | HI | Outcome measures specified include annual measurement of the existing waiting list broken down by county and recommendations to support the implementation and execution of the program. | Caregivers in HI who take care of elderly age 60+, live within the state, need assistance with at least two activities of daily living, or have cognitive impairment | Payment for Caregiver Needs |
| Lifespan Respite Care Program [11] | 2006/2020 | National | Outcome specified include number of states with lifespan respite care programs, caregiver demographics, and effectiveness of entities receiving support. | Caregivers, individuals with special needs, older adults, and military families who require respite care services | Respite Services |
| Maryland Commission on Caregiving [12, 13] | 2016/2017 | MD | No formal outcomes specified. Administrator measures following with assistance from AARP: caregiver concerns, effectiveness and accessibility of caregiver support systems, unmet needs, and additional funding priorities. | Older adults, individuals with disabilities, and their caregivers | Caregiver advocacy |
| Older Americans Act [14] | 1965/2016 | National | Formal outcomes specified include demographic information about care recipient and caregiver, care provided, services rendered or necessary, and expenditure data. | Older adults age 60+ | Care coordination; Research, |
| Older Americans Act; National Family Caregiver Support Program [15 , 16, 17] | 1965/2000 | National | Formal outcome required consisting of an external outcomes report that includes demographic details, perspectives of caregiver role and challenges, services used, impact of caring, and knowledge of programs available. | Unpaid family caregivers of older adults (60+), and grandparents or relatives (55+) caring for children under 18 or with disabilities | Caregiver Training; Counseling, Respite Services |
| Paid Family and Medical Leave [18] | 2016/2023 | 10 States | No formal outcomes specified. There is external research on the impact of family leave. | Working individuals who need time off to care for a new child, a family member with serious health condition | Workplace protections |
| Paid Sick Days [19] | 2015/2022 | 14 States | No formal outcomes specified. There is external research on the impact of family leave. | Private sector employees | Workplace protections |
| Pennsylvania Caregiver Support Act [20] | 1990/2021 | PA | No formal outcomes specified. | Resident caregiver of PA | Caregiver Training; Payment for Caregiver Needs; Counseling Services; Respite Services |
| RAISE Family Caregiver Act [21] | 2017/2018 | National | Outcome evaluation is conducted by individual states and reported to the funding body. External outcome evaluation by Administration for Community Living qualitative case reports from caregivers regarding their experiences and needs. Consideration of expert opinions and expert opinions further guide outcomes. | Caregivers of older adults and individuals with disability, and broader community | Caregiver advocacy |
| Respite for ME [ 22, 23] | 2021/2022 | ME | No formal outcomes specified. Evaluation performed with external research partner, TCARE (Tailored Caregiver Assessment and Referral). To receive the grant funds, TCARE tool is used to assess caregiver needs before, during, and after the grant. The state has contracted with the University of Southern Maine to lead and analyze the results. | ME residents providing care to an family member who is 60+ years old or who has Alzheimer’s Disease or related disorders | Payment for Caregiver Needs; Respite Services |
| TEAM Veteran Caregivers Act [24] | 2019/2020 | National | No formal outcomes specified. | Caregivers of Veterans | Care Coordination |
| MISSION Act [25] | 2018/2018 | National | Formal outcomes specified. Evaluation of the quality of care provided and changes in spending. Further evaluation includes assessment of barriers to care and knowledge of the caregiver. | Veterans eligible for VA healthcare and need access to a wider range of healthcare options | Caregiver Training; Caregiver Advocacy |

**Table S2: Introduced and Enacted Policies**

| **Policy Name** | **Year Introduced** | **Year Passed**  **(**or year amended/most recent state to pass)^a^ | **Location** | **Target Population** | **Benefit(s)** | **Purpose** |
| --- | --- | --- | --- | --- | --- | --- |
| Adult Day Center Enhancement Act [26] | 2021 | NA | National | Individuals living with neurological diseases and conditions, their caregivers | Comprehensive care and support services that maintain or improve functional abilities, prevent complications, promote alternatives to nursing home placement, reduce caregiver strain | Expand and enhance existing adult day programs for younger people with neurological diseases or conditions to support and improve access to respite services for family caregivers who are taking care of such people |
| Alabama Lifespan Respite Resource Network [1] | 2000 | 2016 | AL | Caregivers of individuals with special needs | Provide occasional respite, which strengthens their ability to provide care at home, promotes family stability | Give relief to families and caregivers from the extraordinary and intensive demands of providing ongoing care in the home |
| Alzheimer's Caregiver Support Act [3] | 2021 | NA | National | Individuals with Alzheimer's disease, their caregivers | Provide funding for training and support services to caregivers of people with dementia | Expand training and support services for families and caregivers of individuals living with Alzheimer's disease or a related dementia |
| Alzheimer's State Plan [4] | 2016 | 2016 | WA | Alzheimer's patients, caregivers, and families | Improved coordination and access to quality care and support services | Address the needs of individuals with Alzheimer's disease and their caregivers by implementing a state-specific plan that promotes quality care, enhances support services |
| Autism Family Caregivers Act of 2022 [27] | 2022 | NA | National | Caregivers of individuals with autism spectrum disorder | Provide support and resources to caregiver, including training, respite care, and assistance with daily living activities | Award grants for providing evidence-based caregiver skills training |
| California Family Rights Act [4] | 1993 | 2022 | CA | Family caregiver employees within CA | 12 weeks paid or unpaid leave and medical benefits | Maintain job and medical benefits for employees who need to care for a family member |
| Caregiver Advise, Record, Enable Act [5] | 2016 | 2019 | 42 States | Hospitalized individuals, their caregivers | Hospital discharge planning and reduce readmissions by providing education, training, and support to family caregivers | Improve hospital-to-home transitions and support family caregivers of hospitalized patients |
| Caregiver Assistance Act [28] | 2022 | NA | NJ | NJ family caregiver residents | Provides an individual income tax credit equal to 22.5% of the qualified expenses incurred by a family caregiver to assist a care recipient | Reduce to the financial burden associated with caregiving |
| Caregiver Credit [29] | 2021 | NA | OH | OH family caregiver residents | Provides an individual income tax credit for qualified expenses incurred by a family caregiver to assist a care recipient | Reduce to the financial burden associated with caregiving |
| Caregiver Leave Act [6] | 2016 | 2019 | NM | Family caregiver employees within NM | Allows employees to use their employer sponsored paid or unpaid sick time to care for a loved one | Ensure family caregivers can take necessary time off to perform caregiving tasks |
| Caregivers and Veterans Omnibus Health Services Act of 2010 [7] | 2009 | 2010 | National | Caregivers of post-9/11 veterans and veterans with serious injuries or illnesses | Expanded support services, such as mental health care, respite care, and training | Enhance the quality of life for veterans and their caregivers by improving and expanding healthcare services, providing comprehensive support, counseling, and assistance |
| Comprehensive Care for Alzheimer’s Act [30] | 2021 | NA | National | Individuals with Alzheimer's disease, their caregivers | Enhancing diagnosis, care planning, and coordination of services, as well as providing support | Assess the effectiveness of comprehensive care management services on patient health to unpaid caregivers |
| Dependent Care Assistance Program [8] | 1986 | 2021 | National | Employees with dependent care expenses while at work, like child or elder care | Tax-free dependent care assistance, can contribute up to $5,000 per year | An employee benefit program designed to help reduce the costs associated with the care of a qualifying dependent |
| Essential Caregivers Act [31] | 2021 | NA | TX | Residents of nursing homes and other long-term care facilities,^14^their caregivers | Federally funded facilities with the ability to designate essential caregivers | Ensure that residents have access to the emotional support and critical care provided by their essential caregivers |
| Family Caregiver Act [32] | 2004 | 2004^b^ | IL | IL family caregiver residents | Develops a statewide program to provide information regarding services available, organization of support groups and counseling services, respite care, caregiver training and supplemental services | Systematic support for caregivers to improve access to services and training regarding caregiving needs |
| Family Caregivers Advocacy, Resource and Education Demonstration Program Act of 1986/1987 [33] | 1986 | NA | National | Caregivers of older adults and individuals with disabilities | Access to information, resources, and support services that can enhance their caregiving skills, reduce caregiver burden | Support family caregivers of by providing advocacy, education, information, and referral services, and coordinating these services across the state |
| Family and Medical Leave Act [9] | 1993 | 1993 | National | Employees taking leave for caring for family members | Job protection for up to 12 weeks and the ability to take time off to care for family members with serious health conditions | Balance the needs of employees to take leave for certain family and medical reasons with the business interests of employers |
| Healthy Families Act [34] | 2021 | NA | National | Employees who work for employers with at least 15 employees, including part-time workers | Employees can earn a minimum of seven paid sick days (56 hours) per year | Allowing employees to take time off to care for themselves or a family member without losing income or risking job loss |
| Health Outcomes, Planning, and Education for Alzheimer's Act [35] | 2015 | NA | National | Individuals with Alzheimer's disease, their caregivers | Requiring Medicare to pay for individualized care plans, which provide clear diagnoses, treatment options, and information about available medical and community services | Increase access to information on care and support for newly diagnosed individuals and their families - providing essential support for those facing this devastating, debilitating disease |
| Kupuna Caregivers Program [10] | 2017 | 2018 | HI | Caregivers in HI of people age 60+, need assistance with at least two activities of daily living, or have cognitive impairment | Receive up to $210 / week to help cover the cost of senior care and services | Provides long-term support services, including respite care, transportation, and adult day care, to employed caregivers in Hawaii, with funding directly allocated to service agencies |
| Lifespan Respite Care Program [11] | 2006 | 2020 | National | Caregivers, individuals with special needs, older adults, and military families who require respite care services | Competitive grants of up to $200,000 | Provide temporary relief and support to family caregivers of individuals with special needs or chronic illnesses |
| Maryland Caregiver Tax Credit [36] | 2022 | NA | MD | MD family caregiver residents | Provides an individual income tax credit equal to 30% of the qualified expenses incurred by a family caregiver to assist a family member | Reduce to the financial burden associated with caregiving |
| Maryland Commission on Caregiving [12] | 2016 | 2017 | MD | Older adults, individuals with disabilities, their caregivers | Provide guidance, support, and advocacy for caregivers | Enhance the quality of life for older adults and individuals with disabilities, promote support for caregivers |
| Massachusetts Family Caregiver Tax Credit [37] | 2021 | NA | MA | MA family caregiver residents | Provides an individual income tax credit equal to 50% of the qualified expenses incurred by a family caregiver to assist a care recipient | Reduce to the financial burden associated with caregiving |
| Older Americans Act [14] | 1965 | 2016 | National | Older adults age 60+, their caregivers | Federal grant funds for older Americans | Funding for community and social services, research, and personnel training focusing on older adults.  Development of in home and community long term services  Establishment of caregiver support programs |
| Older Americans Act; National Family Caregiver Support Program (NFCSP) [15] | 1965 | 2000 | National | Unpaid family caregivers of older adults (60+), and grandparents or relatives (55+) caring for children under 18 or with disabilities | Access to information, support, training, counseling, and respite care | Offers grants to states and territories to fund a range of support services that assist caregivers in providing care for older adults in their homes |
| Paid Family and Medical Leave [18] | 2016 | 2022 | 11 States | Working individuals who need time off to care for a new child, a family member with serious health condition | Six to twelve weeks of fully or partially paid leave per year, without the need for accrual | Job-protected and financially-supported time off for individuals to bond with a new child, care for a seriously ill family member, or address their own serious health condition |
| Paid Sick Days [19] | 2015 | 2022 | 14 States | Private sector employees | Allows to take time off work to attend to their own or their family's health needs | Provide paid sick days for those caring for a family member |
| Pennsylvania Caregiver Support Act [20] | 1990 | 2021 | PA | PA family caregiver residents | A range of services including care management, benefits counseling, caregiver education and training, reimbursement for caregiving-related expenses, respite care, and support services | Alleviate the stresses associated with caregiving by focusing on the well-being of the caregiver, access to respite care, addressing the need for formal and informal supports, and providing financial reimbursement for out-of-pocket costs associated with caregiving-related services and supplies |
| RAISE Family Caregiver Act [21] | 2017 | 2018 | National | Caregivers of older adults and individuals with disability, and broader community | Enhanced support services, increased awareness, and a national strategy to assist caregivers | Established the Family Caregiving Advisory Council to provide recommendations on effective models of family caregiving and support, and to improve coordination across federal programs |
| Respite for ME [22] | 2021 | 2022 | ME | ME residents providing care to a family member who is 60+ years old or who has Alzheimer’s Disease or related disorders | Eligible family members who provide care may receive up to $2,000 per year towards eligible services in lieu of accessing respite care services provided by the state | Attempts to alleviate the cost associated with caregiving and allow main residents to stay in their homes for longer |
| Schedules that Work Act [38] | 2019 | NA | National | Low-wage hourly employees facing unpredictable schedules and struggle in managing work, family, education, and health | Promotes fair and flexible scheduling, improving work-life balance, economic stability | Establishes standards for scheduling practices that promote predictability and flexibility for low-wage workers, and allow for necessary schedule changes for caregivers |
| Social Security Caregiver Credit Act of 2021 [39] | 2021 | NA | National | Unpaid family caregivers who provide at least 80 hours of care per month | Add an earnings credit for unpaid time off to their earnings to calculate future Social Security benefits | Provide Social Security earnings credits for caregivers taking unpaid time off work to care for dependent relatives |
| Supporting Our Direct Care Workforce and Family Caregivers Act [40] | 2021 | NA | National | Direct care workers and family caregivers | Provides financial support for direct care workforce training and advancement, and grants to support family caregivers | Improve the quality of care for older adults and individuals with disabilities by providing financial support for direct care workers and family caregivers through grants |
| TEAM Veteran Caregivers Act [24] | 2019 | 2020 | National | Caregivers of veterans | Improves communication of changes, eligibility, or tier reduction to caregivers and their family member; extends benefits when applicable | Improve the administration and provision of caregiver programs for veterans by requiring standardized notifications for clinical determinations, extending benefits after termination for clinical reasons, and identifying caregivers in the health record of the veteran |
| VA MISSION Act [25] | 2018 | 2018 | National | Veterans eligible for VA healthcare and need access to a wider range of healthcare options, their caregivers | Improve access to healthcare through expanded community care options, streamlined appointment scheduling | Access to health care both in VA facilities and in the community, expand benefits for caregivers, and improve the VA's ability to recruit and retain the best medical providers |

^a^NA if never passed.

^b^Passed but not funded at time of analysis, excluded

**Table S3: Evaluative studies and reports**

| **First Author (Year)** | **Policy Name** | **Study Design** | **National/**  **State** | **Outcome Measure (Success Y/N)** | **Vulnerable Populations** | **Costs Examined** | **Study Quality** |
| --- | --- | --- | --- | --- | --- | --- | --- |
| Litzelman (2022) [41] | National Family Caregiver Support Program | Cross-sectional analytic | National | Eligibility requirements allow services available to the majority of caregivers in need (N) | Those who were not eligible for benefits were overrepresented by high-risk subsets of the caregiving population, including low-income caregivers | None | 4 |
| Shugrue (2019) [42] | National Family Caregiver Support Program | Cross-sectional analytic | National | Consistent, standardized outcome evaluations exist to examine the program’s impact on caregivers receiving services and the care recipients that they serve (N) | None | None | 4 |
| Potter (2018) [43] | National Family Caregiver Support Program | Cross-sectional analytic | National | Association between funding and all services used (N); Negative association of policy and unused services (Y) | Caregivers of Black and Hispanic older adults were more likely to report any unused services than all services used (general finding not tied to the policy) | Noted federal funding amounts and how some states supplement with additional funds | 4 |
| Gimm (2016) [44] | California’s Family Rights Act (paid leave law) | Cohort | California | Caregiver mental or physical health (N) | Controlled for SES levels, employment levels, races, ethnicities | Discussed amount of paid leave | 3 |
| Wright (2015) [45] | Caregivers and Veterans Omnibus Health Services Act of 2010 [Caregiver Support Line component] | Cohort | National | Utilization (Y); Satisfaction (Y) | Notes use in rural areas proportional to number of veterans living in rural areas | Notes costs will be evaluated separately | 3 |
| Herrera (2013) [46] | National Family Caregiver Support Program | Cross-sectional analytic | National | Volume of caregiver and other support services use same across races (N); Receiving amount of hours of respite care based on need same across races (N); Living independently due to benefits same across races (N) | When comparing the risk profile of caregivers receiving policy-related services to the general population of seniors, services are reaching the most vulnerable populations according to most risk factors for institutionalization, such as disability, Medicaid coverage, and living alone | Notes potential cost-effectiveness of the program but does not examine | 4 |
| Giunta (2010) [47] | National Family Caregiver Support Program | Cross-sectional analytic | National | State recognize caregivers as both service recipients and provides through counseling and support groups and training (Y) | None | Noted minimal funding level was an implementation barrier | 4 |
| Whittier (2005) [48] | National Family Caregiver Support Program | Cross-sectional analytic | National; California examined for this study | Multiple forms of caregiver service delivery in existence (Y); Caregiver service gaps closed (N) | Areas with a higher than average proportion of their population speaking a language other than English were found to report a greater number of service gaps as did rural areas | Noted caregiver financial assistance in US falls short compared to other countries | 3 |
| AARP (2013)  [49] | Family Medical Leave Policies | Opinion text/case report | National | Employees reported increased ability to care for family member and increased likelihood of returning to work (Y), 88% of workers age 50+ found FMLA to be important to them (Y) | Statistics on low SES groups collected. | None | 5 |
| AARP (2019)  [50] | Older Americans Act | Opinion text/case report | National | Caregivers who receive at least 1 education or support session reported improved confidence (Y), Caregivers who receive 4 or more hours of respite reported reduced caregiver burden (Y) | None | Mentioned services could play a key role in costly interventions | 5 |
| ACL (2021)  [51] | Recognize, Assist, Include, Support, Engage Family Caregivers Act | Opinion text/case report | National | Lack of representation of caregivers in policy and healthcare (N), Variability in how caregiver support is defined across agencies | None | None | 5 |
| Family Caregiver Alliance (2004)  [52] | National Family Caregiver Support Program/ Medicaid Home and Community Support Waivers/ State funded actions | Opinion text/case report | National | Less than half of programs included uniformly assess caregiver needs (N), Respite is most common service offered (Y) | None | Mentioned costs and potential containment strategies | 5 |

References

1. Alabama Lifespan Respite Resource Network.; 2016. Accessed June 11, 2023. <https://alabamarespite.org/about/history/#:~:text=In%202009%2C%20the%20Alabama%20legislature>
2. Geiger BF, O’Neal MR. Determining Provider Needs for Respite Training, Results of an Alabama Survey. SAGE Open. 2014 Dec 18;4(4):215824401456304.
3. Keiser. Alzheimer’s State Plan.; 2014. <https://www.dshs.wa.gov/sites/default/files/legislative/documents/2016%20WA%20Alzheimer%27s%20State%20Plan%20-%20Full%20Report%20Final.pdf>
4. California Family Rights Act.; 1993. <https://leginfo.legislature.ca.gov/faces/billTextClient.xhtml?bill_id=202120220AB1033>
5. Caregiver Advise, Record, Enable (CARE) act. AARP. <https://www.aarp.org/politics-society/advocacy/caregiving-advocacy/info-2014/aarp-creates-model-state-bill.html>
6. Caregiver Leave Act.; 2019. Accessed June 10, 2023. <https://law.justia.com/codes/new-mexico/2020/chapter-10/article-16h/section-10-16h-3/>
7. Caregivers and Veterans Omnibus Health Services Act.; 2010. Accessed June 10, 2023. <https://www.congress.gov/111/plaws/publ163/PLAW-111publ163.pdf>
8. Dependent Care Assistance Program.; 2021. <https://alabamarespite.org/about/history/#:~:text=In%202009%2C%20the%20Alabama%20legislature>
9. Family Medical Leave Act.; 1993. U.S. Department of Labor. https://www.dol.gov/agencies/whd/fmla
10. Hawaii Kupuna Caregivers Program: Eligibility & Benefits. Accessed June 10, 2023. https://www.payingforseniorcare.com/hawaii/kupuna-caregivers
11. Lifespan Respite Care Program.; 2006. ACL Administration for Community Living. https://acl.gov/programs/support-caregivers/lifespan-respite-care-program
12. Hogan. Maryland Commission on Caregiving.; 2017. <https://dhs.maryland.gov/maryland-commission-caregiving/>
13. 2016 Final Report Task Force on Family Caregiving and Long Term Supports [Internet]. ARCH National Respite Network. 2016. Available from: https://archrespite.org/library/2016-final-report-of-the-task-force-on-family-caregiving-and-long-term-supports/
14. Older Americans Act.; 1965. ACL Administration for Community Living. <https://acl.gov/about-acl/authorizing-statutes/older-americans-act>
15. National Family Caregiver Support Program.; 2000. ACL Administration for Community Living. <https://acl.gov/programs/support-caregivers/national-family-caregiver-support-program>
16. Metzelthin SF, Verbakel E, Veenstra MY, van Exel J, Ambergen AW, Kempen GIJM. Positive and negative outcomes of informal caregiving at home and in institutionalised long-term care: a cross-sectional study. BMC Geriatr. 2017;17(1):232. doi:10.1186/s12877-017-0620-3
17. Avison C, Brock D, Campione J, Hassell S, Ritter R, Severynse J, et al. Outcome Evaluation of the National Family Caregiver Support Program. Rockville, MD: Westat; 2018 Dec
18. Paid Family and Medical Leave. U.S. Department of Labor. Published 2022. <https://www.dol.gov/agencies/wb/featured-paid-leave>
19. Paid Sick Days.; 2022. <https://nationalpartnership.org/wp-content/uploads/2023/02/current-paid-sick-days-laws.pdf>
20. Pennsylvania Caregiver Support Act.; 1990. <https://www.aging.pa.gov/publications/policy-procedure-manual/Documents/App%20E%203%20-%20Act%2020%20of%202021.pdf#:~:text=Establishing%20a%20program%20to%20assist%20persons%20who%20bear,disabilities.%20%28Title%20amended%20June%2011%2C%202021%2C%20P.L.52%2C%20No.20%29>
21. Harper. RAISE Family Caregiver Act.; 2018. <https://acl.gov/programs/support-caregivers/raise-family-caregiving-advisory-council>
22. Respite for ME. <https://nashp.org/respite-for-me-maines-new-respite-care-pilot-for-family-caregivers/>
23. Hodges K, Fox-Grage W. Respite for ME: Maine’s New Respite Care Pilot for Family Caregivers [Internet]. NASHP. 2022. Available from: <https://nashp.org/respite-for-me-maines-new-respite-care-pilot-for-family-caregivers/>
24. TEAM Veteran Caregivers Act.; 2020. Accessed June 12, 2023. <https://biggs.house.gov/media/press-releases/congressman-biggs-introduces-team-veteran-caregivers-act>
25. VA MISSION Act.; 2018. Congress.gov. <https://www.congress.gov/bill/115th-congress/senate-bill/2372/text>
26. Lee. Adult Day Center Enhancement Act.; 2021. <https://www.govinfo.gov/app/details/BILLS-117hr253ih>
27. Meng. Autism Family Caregivers Act of 2022.; 2022. <https://www.govinfo.gov/app/details/BILLS-117hr6783ih>
28. Lampitt. Caregiver Assistance Act.; 2022. <https://legiscan.com/NJ/bill/A1802/2022>
29. Russo. Caregiver Credit.; 2021. Ohio House of Representatives. Accessed June 12, 2023. <https://ohiohouse.gov/members/c-allison-russo/news/reps-russo-boyd-reintroduce-the-caregiver-expenses-tax-credit-105322>
30. Higgins. Comprehensive Care for Alzheimer’s Act.; 2021. <https://www.govinfo.gov/app/details/BILLS-117hr2517ih>
31. Essential Caregivers Act.; 2021. <https://essentialcaregivermovement.org/about-3733>
32. Family Caregiver Act.; 2004. <https://www.ilga.gov/legislation/ilcs/ilcs3.asp?ActID=2610&ChapterID=31>
33. Glenn. Family Caregivers Advocacy, Resource, and Education Demonstration Program Act of 1987.; 1987. <https://www.congress.gov/bill/100th-congress/senate-bill/449/cosponsors?s=1&r=56&overview=closed>
34. Murray. Healthy Families Act.; 2021. <https://nationalpartnership.org/wp-content/uploads/2023/02/the-healthy-families-act-fact-sheet.pdf>
35. Smith. Health Outcomes, Planning, and Education (HOPE) for Alzheimer’s Act.; 2015. <https://alzimpact.org/improving_hope_for_alzheimers_act#:~:text=Signed%20into%20law%20in%20December>
36. Benson. Maryland Caregiver Tax Credit.; 2022. <https://mgaleg.maryland.gov/mgawebsite/Legislation/Details/sb0660?ys=2022RS>
37. Linsky. Massachusetts Family Caregiver Tax Credit.; 2021. <https://malegislature.gov/Bills/192/H2979/BillHistory>
38. Schedules That Work Act.; 2019. Accessed June 12, 2023. <https://www.warren.senate.gov/newsroom/press-releases/warren-delauro-reintroduce-the-schedules-that-work-act#:~:text=The%20Schedules%20That%20Work%20Act%20curbs%20these%20harmful%20practices%20by>
39. Murphy. Social Security Caregiver Credit Act.; 2021. Published May 28, 2021. Accessed June 12, 2023. <https://www.murphy.senate.gov/newsroom/press-releases/murphy-schneider-meng-introduce-legislation-to-help-caregivers-receive-social-security-credit>
40. Kaine. Supporting Our Direct Care Workforce and Family Caregivers Act.; 2021. <https://www.kaine.senate.gov/press-releases/kaine-leads-introduction-of-bill-to-support-direct-care-workforce-and-family-caregivers>
41. Litzelman K, Harnish A. Caregiver Eligibility for Support Services: Correlates and Consequences for Resource Utilization. J Appl Gerontol Off J South Gerontol Soc. 2022;41(2):515-525. doi:10.1177/0733464820971134
42. Shugrue N, Kellett K, Gruman C, et al. Progress and Policy Opportunities in Family Caregiver Assessment: Results From a National Survey. J Appl Gerontol. 2019;38(9):1319-1341. doi:10.1177/0733464817733104
43. Potter AJ. Factors Associated With Caregivers’ Use of Support Services and Caregivers’ Nonuse of Services Sought. J Aging Soc Policy. 2018;30(2):155-172. doi:10.1080/08959420.2017.1414539
44. Gimm G, Yang YT. The Effect of Paid Leave Laws on Family Caregivers for the Elderly. Ageing Int. 2016;41(2):214-226. doi:10.1007/s12126-016-9242-x
45. Wright P, Malcolm C, Hicken B, Rupper R. The VA Caregiver Support Line: A Gateway of Support for Caregivers of Veterans. J Gerontol Soc Work. 2015;58(4):386-398. doi:10.1080/01634372.2015.1019168
46. Herrera AP, George R, Angel JL, Markides K, Torres-Gil F. Variation in Older Americans Act Caregiver Service Use, Unmet Hours of Care, and Independence Among Hispanics, African Americans, and Whites. Home Health Care Serv Q. 2013;32(1):35-56. doi:10.1080/01621424.2012.755143
47. Giunta N. The National Family Caregiver Support Program: A Multivariate Examination of State-Level Implementation. J Aging Soc Policy. 2010;22(3):249-266. doi:10.1080/08959420.2010.485523
48. Whittier S, Scharlach A, Dal Santo TS. Availability of Caregiver Support Services: Implications for Implementation of the National Family Caregiver Support Program. J Aging Soc Policy. 2005;17(1):45-62. doi:10.1300/J031v17n01_03
49. Feinberg L. Keeping Up with the Times: Supporting Family Caregivers with Workplace Leave Policies. Washington, DC: AARP Public Policy Institute; 2013 p. 1–18
50. Ujvari K, Fox-Grage W, Houser A. Spotlight: Older Americans Act. Washington, DC: AARP Public Policy Institute; 2019 Feb.
51. Recognize, Assist, Include, Support, & Engage (RAISE) Family Caregivers Act Initial Report to Congress. Administration for Community Living; 2021.
52. Feinberg L, Newman S, Gray L, Kolb K. The State of the States in Family Caregiver Support: A 50-State Study. Family Caregiver Alliance; 2004 Nov.
